# Reduced Serum Levels of Soluble Interleukin-15 Receptor α in Schizophrenia and Its Relationship to the Excited Phenotype

**DOI:** 10.3389/fpsyt.2022.842003

**Published:** 2022-03-09

**Authors:** Yi He, Qijing Bo, Zhen Mao, Jian Yang, Min Liu, Haixia Wang, Abba J. Kastin, Weihong Pan, Chuanyue Wang, Zuoli Sun

**Affiliations:** ^1^Beijing Key Laboratory of Mental Disorders, The National Clinical Research Center for Mental Disorders, Beijing Anding Hospital, Capital Medical University, Beijing, China; ^2^Advanced Innovation Center for Human Brain Protection, Capital Medical University, Beijing, China; ^3^Pennington Biomedical Research Center, Baton Rouge, LA, United States; ^4^BioPotentials Consult, Sedona, AZ, United States

**Keywords:** IL-15Rα, schizophrenia, depression, excited phenotype, pre-pulse inhibition

## Abstract

Our previous studies documented that interleukin-15 receptor α (IL-15Rα) knockout (KO) mice exhibited hyperactivity, memory impairment, and desperate behavior, which are core features of schizophrenia and depression. Due to the overlapping symptomology and pathogenesis observed for schizophrenia and depression, the present study attempted to determine whether IL-15Rα was associated with the risk of schizophrenia or depression. One hundred fifty-six participants, including 63 schizophrenia patients, 29 depressive patients, and 64 age-matched healthy controls, were enrolled in the study. We investigated the circulating levels of soluble IL-15Rα and analyzed potential links between the IL-15Rα levels and clinical symptoms present in schizophrenia or depressive patients. We observed reduced serum IL-15Rα levels in schizophrenia patients, but not depressive patients compared with controls. Moreover, a significant negative association was observed between the circulating IL-15Rα levels and excited phenotypes in the schizophrenia patients. The IL-15Rα KO mice displayed pronounced pre-pulse inhibition impairment, which was a typical symptom of schizophrenia. Interestingly, the IL-15Rα KO mice exhibited a remarkable elevation in the startle amplitude in the startle reflex test compared to wild type mice. These results demonstrated that serum levels of soluble IL-15Rα were reduced in schizophrenia and highlighted the relationship of IL-15Rα and the excited phenotype in schizophrenia patients and mice.

## Introduction

Psychiatric disorders, such as schizophrenia and major depressive disorder (MDD), result from complex interactions between genetic and environmental factors that lead to developmental or neurological impairment (1, 2). The overlapping clinical phenotypes and genetic associations observed with various psychiatric disorders suggest a potential for shared elements of their disease etiology (3–6).

Considerable evidence indicates that disruption of the immune system due to inflammation has a critical role in psychiatric disorders, including schizophrenia and depression (3, 4, 7). Recent data indicate the presence of typical inflammation responses in schizophrenia and depression (3, 8–10). Epidemiological studies have revealed that the presence of an autoimmune disease is a high-risk factor for psychiatric disorders (11, 12). A series of genome-wide association studies (GWAS) of schizophrenia confirmed that the immune system is involved in the pathogenesis of schizophrenia and depression, including the HLA gene (a complement gene) and the interleukin gene family (13–15). Furthermore, overactivation of microglia in the brain and changes in lymphocyte functions in the systemic circulation also have been reported in previous studies (3, 16). Increases in pro-inflammatory factors (e.g., IL-1, IL-6, and TNF-α) or reduction of anti-inflammatory factors (e.g., IL-4 and IL-10) also were found in the serum of patients with mental disorders (17–19). In addition, animals with overactive inflammatory cytokines displayed schizophrenia- or depressive-like behaviors, and the cytokines induced neuronal loss and synaptic impairment (20, 21), which are common characteristics observed in schizophrenia and depression.

Although the relationship between the immune system and psychiatric diseases has been studied extensively, the underlying mechanisms of action are still unclear. The functions of immune molecules *in vivo* are complex and overlap to orchestrate complex behaviors (22, 23). Essential questions still need to be answered, including, are there additional critical immune molecules that have significant roles in schizophrenia and depression? Also, are there differences among immune molecules that mediate functions between schizophrenia and depression?

Our previous data indicated that the receptor subunit of IL-15, IL-15Rα, is an important immune molecule that maintains normal nervous system functions. We reported that IL15Rα knockout (KO) mice displayed hyperactivity in the open-field test, reduced social interactions in the three-chamber test, defects in learning ability in the T-maze, and depressive-like behavior in the forced immobility test (24–26). Collectively, these results indicate that IL-15Rα plays an important role in schizophrenia and depression. Furthermore, we found a rare mutation in the *IL-15RA* gene that impeded IL-15Rα intracellular signal transduction in schizophrenia patients (27). As the ligand of IL-15Rα, IL-15 is a cytokine especially poised to have a pivotal role in CNS organization, and it also has been reported to be a biomarker of schizophrenia and depression (28–30). In fact, *IL-15RA* mRNA is widely expressed in the brain, including in microglia, astrocytes, and neurons, and it exhibits a major signaling role in regulating brain homeostasis and development (31–33). For example, IL-15/IL-15R could reduce the serotonin transmission which led to the depressive phenotypes in mice (25). Previous study also demonstrated that IL-15/IL-15R resisted environment stress through Akt signaling transmission (34). IL-15Rα is ubiquitously produced in the brain as well as other organs; it activates multiple intracellular kinases through a trimeric receptor complex consisting of its specific receptor IL-15Rα and co-receptors interleukin 2 receptor β and interleukin 2 receptor γ (32, 35, 36). Both IL-15 and IL-15Rα have multiple splice variants and exist in different subcellular compartments (37–40), suggesting that IL-15 signaling exhibits vital functions in neurodevelopment. IL-15 also can signal through IL-15Rα in the absence of other members of the trimeric complex, suggesting that this particular monomeric signaling unit has specific functions (41). We have observed that IL-15Rα KO mice display defects in GABAergic and serotonergic transmission, contributing to the phenotypes of schizophrenia and depression (24–26). The deficits observed in IL-15Rα KO mice are greater than those seen in mice without interleukin 2 receptor β (42), brain-derived interleukin 2 (43), or interleukin 2 receptor γ co-receptor (42, 44).

In this study, we investigated whether IL-15Rα was associated with the risk for psychiatric disorders by comparing the serum-soluble IL-15Rα levels in patients with schizophrenia or depression and healthy controls. We also explored the association of IL-15Rα expression with psychiatric symptoms in patients. We observed whether similar psychiatric symptoms were shown in mice with depletion of IL-15Rα. When combined with our previous results from animal experiments, this study provided clinical evidence of the role of IL-15Rα in psychiatric diseases.

## Materials and Methods

### Subjects

This study was approved by the Independent Ethics Committee (IEC) of the Beijing Anding Hospital, Capital Medical University, Beijing, China (2015127FS-2). Each subject in the present study provided informed written consent.

A diagnosis of schizophrenia or depression was confirmed by administering the Structured Clinical Interview for DSM-IV (SCID) by experienced psychiatrists. The inclusion criteria for all patients were aged 16 to 60 years, formal education ≥ 9 years, and total scores of the Young Mania Rating Scale (YMRS) ≤ six. The exclusion criteria for all patients were comorbidity with other psychiatric disorders, displayed severe suicidal tendencies, currently substance dependent or had a history of substance dependence, had severe physical diseases, including neurological, cardiovascular, hepatic, respiratory, or renal diseases, and were pregnant or breastfeeding.

In this study, we recruited age- and gender-matched healthy participants with no history of psychosis or cognitive impairment diseases, such as mild cognitive impairment and Alzheimer’s disease. Each healthy individual underwent a psychiatric interview by experienced clinicians using the SCID to exclude the presence of any psychiatric disorders. Healthy controls were excluded when the following situations were encountered: including had any lifetime DSM-IV psychiatric disorder, had severe physical diseases, such as neurological, cardiovascular, hepatic, respiratory, or renal diseases, had a family history of psychiatric disease, currently substance dependent or had a history of substance dependence, were pregnant or breastfeeding.

### Clinical Assessments

Clinical assessments of the patients were carried out by two psychiatrists who had more than 5 years of experience in clinical practice. The psychiatrists had received consistency training, and the assessments had a good consistency (Cohen’s = 0.8). The two psychiatrists were blind to the clinical status and treatment conditions of the participants. Baseline sociodemographic characteristics were collected using a questionnaire specifically designed for the present study. For schizophrenia, the severity of the schizophrenia symptoms was evaluated using the Positive and Negative Syndrome Scale (PANSS), which included 30 items. Subscores were used to evaluate the different phenotypes according to the five-factor dimensional model of schizophrenic symptoms (positive, negative, excitement, depression, and cognitive) (45). For depression, the patients were rated using the Hamilton Depression Scale (HAMD), and the severity of depression symptoms was rated. The HAMD-17 is the most widely utilized instrument for evaluating depressive symptoms. The HAMD is a 17-item clinician-administered assessment scale that is acutely sensitive to changes in patients with severe depression as it emphasizes the physical symptoms of depression (46). In addition, the severity of anxiety symptoms was assessed using Hamilton Anxiety Scale (HAMA).

Due to the cognitive impairment that is commonly found in schizophrenia patients, the MATRICS Consensus Cognitive Battery (MCCB) was used to evaluate the cognitive function of the schizophrenic patients in this study. The MCCB, which is sensitive to cognition as assessed in clinical trials, is comprised of 10 standardized measures used to calculate cognition in seven domains, including the speed of processing, attention/vigilance, working memory, verbal learning and memory, visual learning and memory, reasoning and problem-solving skills, and social cognition (47). Also, a total score was calculated across the seven domains to evaluate the global cognitive function.

### Detection of Serum Soluble Interleukin-15 Receptor α Levels

Whole blood was collected from each subject into EDTA tubes after their clinical assessment, which occurred between 8:00 a.m. and 15:00 p.m. The serum was harvested after centrifugation at 3,000 rpm for 10 min at room temperature. The serum soluble IL-15Rα levels in all participants were assessed using a solid-phase sandwich enzyme-linked immunosorbent assay (ELISA) (CUSABIO, Wuhan, China). The assay sensitivity was 7.8 pg/ml, and the intra- and inter-assay coefficients of variation were < 10%. To avoid inter-test variations, duplicate measurements were made simultaneously, using the same ELISA kit.

### Interleukin-15 Receptor α Knockout Mice

IL15Rα KO mice were purchased from The Jackson Laboratory (003723). This line of homozygous mice was initially produced and donated by Dr. Averil Ma’s lab to The Jackson Laboratory. Exons 2 and 3 were replaced in these mice with a neomycin resistance gene cassette ligated to a thymidine kinase gene cassette, resulting in the complete loss of production of full-length IL15Rα in the embryos (31). The B6.129 strain had been backcrossed with C57 (B6) mice in our laboratory for at least six generations. The last generation of heterozygote mice was mated, and the resulting homozygous KO and wild type (WT) offspring of these mice were used in the experiments. The genotype blotting image was shown in Figure 2A. Nine KO mice and sixteen WT mice were used in this study. The animal experiment was approved by the Animal Use and Care Committee at Capital Medical University. We followed the local ethical and safety rules for the humane use of animals in research.

### Pre-pulse Inhibition Tests for Mice

Mice were maintained using a 12 h light-dark cycle and group housing, with food and water access *ad libitum*. The IL-15Rα KO and WT mice were tested for PPI to acoustic startle responses, a behavioral measure that is considered to be a schizophrenic endophenotype. The acoustic startle response was measured using the MED startle reflex system (Med Associates, St. Albans, VT, United States) in IL-15Rα KO and WT males at four m of age (n = 9-16/group). The test was performed between 9:30 and 11:30 a.m., and the mice were tested in a random order in both groups. After five min of acclimatization, each mouse underwent a habituation block of 30 trials spaced 20 s apart. Each trial consisted of 20 ms of an acoustic signal with an intensity of 105 dB. This was followed by a second block of pre-pulse stimulation with 40 trials spaced 20 s apart. Each trial was preceded by a 75 dB pre-pulse of four ms occurring either 30 or 100 ms before the 20 ms 105 dB startle pulse (Figure 2B). The PPI was calculated using the following formula: PPI% = (1 – prepulse plus startle amplitude/startle only amplitude) × 100.

### Statistical Analysis

The data were analyzed using SPSS statistical software (version 20.0, SPSS Inc., Chicago, IL, United States). Descriptive statistics were used to present the sample characteristics as means ± standard deviation (SD). Comparisons of demographic and IL-15Rα levels among the groups were performed using one-way analysis of variance (ANOVA) followed by *post hoc* Bonferroni multiple comparison tests. Gender and family history were assessed using the chi-square test. The analysis of covariance followed by *post hoc* Bonferroni multiple comparison tests was used to eliminate the influence of antipsychotics when comparing IL-15Rα levels among the three groups. A multiple linear regression analysis was used to evaluate the association between serum IL-15Rα levels and clinical symptoms or cognitive function. The comparison of PPI was analyzed with an independent *t*-test between IL-15Rα KO mice and wild type mice. A two-tailed statistical significance level was set at *p* < 0.05 for all tests.

## Results

### Demographic and Clinical Characteristics of the Participants

One hundred and fifty-six participants, including 63 schizophrenia patients, 29 depressive patients, and 64 age- and sex-matched healthy controls, were enrolled in this study (Table 1). The time from the first appearance of psychotic symptoms to enrollment in the study (psychotic symptom duration) was 25.60 months (SD = 25.11) in schizophrenia patients and 59.86 months (SD = 58.17) in depressive patients. For the schizophrenia patients, 20 individuals were not taking antipsychotics at the time of enrollment, and 43 patients were taking antipsychotics, including risperidone, clozapine, olanzapine, quetiapine, paliperidone, and amisulpride. Eight depressive patients were not taking antipsychotics at the time of enrollment, and 21 patients were taking various psychotropics, including antidepressants (escitalopram and duloxetine), antipsychotics (olanzapine, aripiprazole, and quetiapine), and mood stabilizers (lithium and valproic acid). The doses equivalent to olanzapine were shown in Table 1 (48).

**TABLE 1:** Demographic variables and clinical characteristics in healthy controls, schizophrenic patients, and depressive patients.

	Healthy	Schizophrenia	Depression	F/χ^2^	*p*
Number	64	63	29		
Age (years)	26.03 ± 3.94	25.08 ± 6.60	27.83 ± 4.63	2.675^a^	0.072
Gender (M/F)	40/24	28/35	17/12	4.419^b^	0.11
Education (years)	13.72 ± 2.84	12.84 ± 3.28	13.10 ± 3.45	1.274^c^	0.283
Duration of illness (months)	–	25.60 ± 25.11	59.86 ± 58.17	–	–
Family history (No/Yes)	25/0	49/13	24/5	6.063^b^	0.048*
Antipsychotics (Yes/No)	–	43/20	21/8		
Olanzapine equivalents (mg)	–	2.59 ± 5.00	1.58 ± 4.98	–	–
IL-15Rα concentration (pg/ml)	195.13 ± 110.51	150.12 ± 99.57	208.36 ± 101.72	4.304^c^	0.015*
**PANSS scores**					
*Negative*	–	31.00 ± 10.10	–	–	–
*Positive*	–	19.49 ± 3.36	–	–	–
*Excited*	–	13.94 ± 4.28	–	–	–
*Depressive*	–	9.14 ± 3.34	–	–	–
*Cognitive*	–	13.83 ± 3.54	–	–	–
*Total*	–	87.40 ± 13.04	–	–	–
MCCB total scores	–	35.15 ± 6.45	–	–	–
HAMD-17 total scores	–	–	12.07 ± 8.28	–	–
HAMA total scores	–	–	13.55 ± 17.47	–	–

M, male; F, female. Values represent means ± S.D. *p < 0.05. ^a^Non-parametric Kruskal-Wallis test. ^b^Chi-square test. ^c^Analysis of variance.

### Reduction of Serum Interleukin-15 Receptor α Levels Was Observed in Schizophrenia Patients and Not Depressive Patients

**FIGURE 1:**
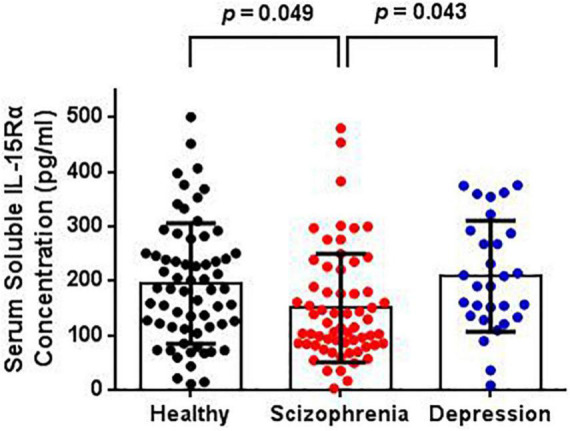
Comparison of serum soluble IL-15Rα levels in schizophrenia patients, depression patients, and healthy controls. The serum soluble IL-15Rα levels were significantly decreased in schizophrenia patients than that in depressive patients or controls.

The serum IL-15Rα levels in each group are shown in Table 1 and Figure 1. The average serum IL-15Rα levels were 150.12 (SD = 99.57), 208.36 (SD = 101.72), and 195.13 (SD = 110.51) pg/ml in the schizophrenia patients, depressive patients, and healthy controls, respectively. As shown in Figure 1, a significant decrease in serum IL-15Rα levels was present in schizophrenia patients compared with healthy controls (*p* = 0.049) or depressive patients (*p* = 0.043). However, no significant differences were observed in the serum IL-15Rα levels between depressive patients and controls (*p* > 0.05).

**FIGURE 2:**
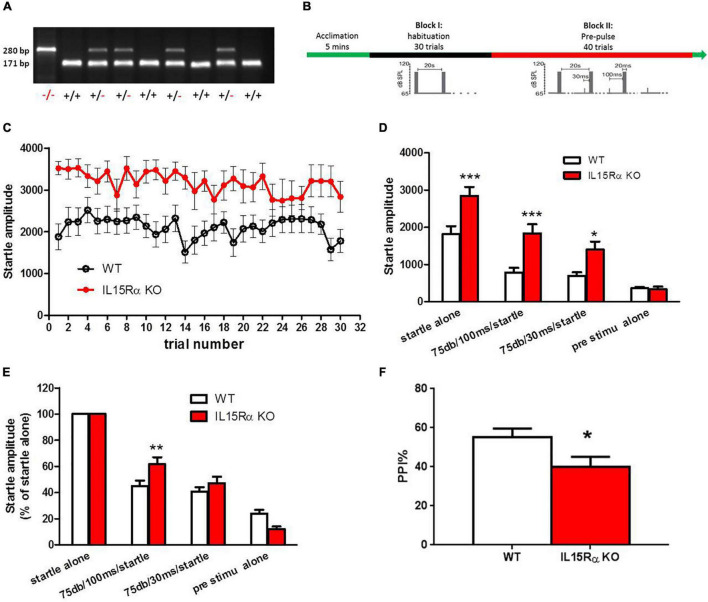
IL-15Rα Knockout mice displayed obvious PPI deficit. **(A)** Blotting image of genotyping in mice. **(B)** Experimental design of PPI. **(C)** The startle amplitude of IL-15Rα KO mice or wild types in PPI test. **(D)** Comparions of startle amplitude between IL-15Rα KO mice and wild types in PPI test. **(E)** Comparisons of startle amplitude with different kinds of pre-stimulation pattern. **(F)** Comparion of PPI at 75 db with a 100 ms interval between IL-15Rα KO mice and wild types. *n* = 9-16 per group. *p < 0.05, **p < 0.01, ***p < 0.001 vs. wild types.

To eliminate the influence of confounding factors, such as antipsychotics on serum IL-15Rα levels in patients, the analysis of covariance was used in this study. There was a significant difference in serum IL-15Rα levels among the three groups (*F* = 4.547, *p* = 0.015). Consistent with the above result, significant decreases of IL-15Rα levels in schizophrenia patients were also found compared with healthy controls (*p* = 0.031) or depressive patients (*p* = 0.016) when controlling for age, sex and doses equivalent to olanzapine. Moreover, we found no significant difference in IL-15Rα levels between drug-free and drug-treated schizophrenia patients (Supplementary Table 1).

### Association of Serum Interleukin-15 Receptor α and the Excited Component in Schizophrenia

A multiple linear regression analysis was used to evaluate the relationship between the clinical symptoms and serum IL-15Rα concentrations (Table 2). For schizophrenia patients, a remarkable negative correlation existed between the levels of IL-15Rα and the excited component (*B* = −11.765, *t* = −2.603, *p* = 0.014). However, no significant association was observed between the IL-15Rα levels and other clinical phenotype or the cognitive function in depressive patients (Table 2 and Supplementary Table 2).

**TABLE 2:** Associations of the serum IL-15Rα levels and phenotypes in schizophrenia patients.

	*B*	Standard coefficient	*t*	*p*
**Schizophrenia**				
Age	3.132	0.202	1.021	0.315
Gender	−40.034	−0.190	−0.921	0.365
**PANSS scores**				
*Negative*	−0.494	−0.048	−0.205	0.839
*Positive*	3.704	0.126	0.661	0.513
*Excited*	−11.765	−0.506	−2.603	0.014
*Depressive*	8.009	0.250	1.361	0.184
*Cognitive*	8.543	0.299	1.252	0.220
MCCB total scores	−0.854	−0.052	−0.272	0.787
**Depression**				
Age	−3.396	−0.094	−0.661	0.515
Gender	−50.459	−0.237	−1.157	0.259
HAMD-17 total scores	−0.277	0.002	−0.092	0.927
HAMA total scores	−0.962	−0.162	−0.617	0.543

*PANSS, Positive and Negative Syndrome Scale; MCCB, MATRICS consensus cognitive battery; HAMD, Hamilton Depression Scale; HAMA, Hamilton Anxiety Scale.*

### Interleukin-15 Receptor α Knockout Mice Showed a Schizophrenia-Like Phenotype

The results described above indicated that the reduced serum levels of IL-15Rα might have a close relationship with the excited phenotype of schizophrenia. To explore the direct relationship of IL-15Rα with the schizophrenia-like phenotypes in schizophrenia, we established the IL-15Rα KO mice. We found a remarkable elevation in the startle amplitude regardless of whether prestimulation occurred or was absent in IL-15Rα KO mice compared to wild type mice (Figures 2C,D). Furthermore, the IL-15Rα KO mice showed a significant decrease in PPI, which was consistent with our published data that showed reduced PPI in schizophrenia patients (Figures 2E,F) (49).

## Discussion

The present study investigated potential connections between IL-15Rα levels in serum and the risk of schizophrenia and depression. Three findings were obtained in our study. (1) Patients with schizophrenia but not depression showed significant reductions in serum IL-15Rα levels compared to healthy controls. (2) A significant negative correlation was found between serum IL-15Rα concentrations and the excited component of the PANSS scores in schizophrenia patients. (3) A remarkable terrified response in the IL-15Rα KO mice was observed in the PPI test, and the KO mice displayed obvious PPI impairment.

Several published reports have confirmed that IL-15Rα insufficiency results in multiple disorders, including but not limited to immunodeficiency, skeletal muscle variations, and a range of neurological symptoms (24, 50–54). The presence of inflammation is an essential etiological hypothesis in psychiatric diseases, including schizophrenia and depression, and we observed alterations in the serum levels of IL-15Rα in patients with schizophrenia and depression in this study. Interestingly, we found a significant reduction in IL-15Rα levels in schizophrenia but no changes in depressive patients. Notably, we also found a distinct decrease of IL-15Rα in schizophrenia patients compared to depressive patients. These observations correlated with our previous study that showed variants of the *IL-15RA* gene are associated with schizophrenia using *IL-15RA* exon sequencing (27).

Several reasons might be related to the different expression of IL-15Rα in serum between schizophrenia patients and depressive patients. First, although clustering analysis showed that schizophrenia and MDD share similar patterns of inflammatory changes, genome-wide genetic studies (7) have reported evidence that supports the presence of a more extensive inflammatory response in schizophrenia than depression. For example, patients with schizophrenia exhibited higher levels of inflammatory markers in their circulatory system than depressive patients or healthy controls (55). A recent postmortem study using transcriptional profiling revealed that samples from schizophrenia patients showed increased expression of transcripts associated with inflammation across all brain regions examined (3). These findings were not evident in patients with bipolar disease or depression or rat brains following chronic dosing with antipsychotic drugs (3). Second, in addition to exhibiting altered inflammatory responses, IL-15Rα KO mice exhibit several unique alterations in brain development and function without increased mortality (31, 32). IL-15Rα also was involved in blood-brain barrier development, affecting cellular permeability (56). Based on the Developing Human Transcriptome database^1^, we observed that IL-15Rα is not expressed in the brain before birth but rises sharply after birth, reaching a peak at the age of 2 and 3, and then high levels of expression are maintained. These data suggested that the function of IL-15Rα is essential during early postnatal brain development. Considering the close relationship between impairment of brain development and schizophrenia, we speculated that a greater reduction in IL-15Rα might accompany schizophrenia. Third, most of the patients were taking antipsychotics (68% in schizophrenia and 72% in depression), so the influence of antipsychotics on IL-15Rα levels should be considered. Although no study so far illustrated the effect of antipsychotics on IL-15Rα, several data showed the significant changes in circulating IL-15 levels in schizophrenia patients than that in controls, yet no significant change of IL-15 levels were found in patients prior and pose-treated with 6-8 weeks of antipsychotics (29, 57). Moreover, there was no remarkable difference in IL-15Rα levels in schizophrenia patients treated with or without antipsychotics before enrollment (Supplementary Table 1). Our covariance analysis controlling for antipsychotics also found a significant decrease in schizophrenia patients while not in patients with depression. These suggested that antipsychotics might have limited effect on circulating IL-15Rα levels.

The underlying reason for reduced soluble IL-15Rα in the plasma of schizophrenia patients was elusive. It was plausible that the decrease in the membrane protein IL-15Rα, the source of soluble receptors, contributed to the reduction in soluble IL-15Rα. Another reason might be a decrease in cleaved enzyme activity, which removes the soluble IL-15Rα from the membrane protein IL-15Rα, such as TACE/ADAM17 (58, 59). This possibility was supported by a previous study in which schizophrenia patients exhibited significantly higher levels of *ADAM17* mRNA in whole blood compared to controls (60). However, another postmortem study by Marballi et al., demonstrated that ADAM17 protein levels in schizophrenia patients were significantly higher than controls in BA9 (61). These data suggested that different alterations in IL-15Rα levels might be found between the peripheral systems and brain, especially with respect to the subregions of the brain.

We analyzed the association of IL-15Rα levels and clinical symptoms in the schizophrenia patients further to assess the relationship between IL-15Rα and schizophrenia phenotypes. We found that serum IL-15Rα levels showed a considerable, negative correlation with the excited phenotype in schizophrenia patients. This result was verified by our animal study, which indicated that the IL-15Rα KO mice displayed a distinct PPI impairment, which was considered the endophenotype of schizophrenia. It was noteworthy that our previous data indicated that the IL-15Rα KO mice exhibited hyperactivity in the open-field test (54). The hyperexcitability induced by reduced IL-15Rα levels or deficiency could be mediated by synaptic redundancy or insufficient GABAergic transmission. Huang et al., demonstrated that IL-15 treatment of rat neural stem cells reduced neurite outgrowth in differentiating neurons, suggesting that insufficient IL-15Rα signaling might induce hyperexcitability in neurons through excessive neurites (62). Our previous work indicated that reduced expression of IL-15Rα resulted in the reduced expression of GABA in hippocampal homogenates, resulting in enhanced excitatory transmission in the nervous system (24). In addition, the IL-15Rα KO mice also displayed a higher startle reflex, which manifested as the increased startle amplitude than wild type in this study. The reduced GABA or glycine transmissions, or the increased glutamate transmission might contribute to excessive startle response, also suggesting that IL-15Rα was associated with increased excitability (63–66). These data promoted the viewpoint that the reduced IL-15Rα not only resulted in schizophrenia, but also contributed to the excitatory phenotype in schizophrenia.

Although few reports have described a role for IL-15Rα in schizophrenia, several studies have demonstrated that the underlying mechanism might be related to abnormal lipid and energy biosynthesis and metabolism. In fact, IL-15 has attracted considerable attention as a potential regulator to prevent and/or treat obesity and metabolic dysfunction (67). IL-15 might reduce triacylglycerol absorption and has been shown to inhibit lipid deposition in murine 3 T3-L1 preadipocytes (68). Moreover, an earlier study using fluorescence resonance energy transfer and confocal microscopy reported that IL-2 and IL15Rα were co-expressed in a supramolecular receptor cluster in lipid rafts of T cells, indicating that these molecules might have essential functions in lipid metabolism (69). The global IL-15Rα KO mice exhibited lower body fat levels, which provided direct evidence of a role of IL-15Rα in lipid regulation (54, 70, 71). Our recently published work with RNA-seq of the cortex and hippocampus of IL-15Rα KO mice identified three functional clusters, including respiratory chain and electron transport, regulation of steroid processes, and skeletal muscle development (72). These results highlight the important role of IL-15Rα in lipid and energy metabolism, which might be mediated through STAT3 phosphorylation (27, 62).

Several limitations should be noted in this study. First, the sample size of each group was small, especially the MDD group. The disequilibrium might affect the reliability of results. Second, the confounding effect of antipsychotics on the changes in serum IL-15Rα levels in patients could not be ruled out although we did not observe any effect of antipsychotic treatment on serum IL-15Rα levels in schizophrenia patients (Supplementary Table 1). Cohort studies of antipsychotics therapy are needed in the following study. In addition, the blood collection without fasting also might be a confounding factor on serum IL-15Rα levels. Third, the cognitive function was not evaluated in depressive patients in this study.

Therefore, although this was a pilot study, when the data were considered collectively, they revealed a definitive reduction in serum IL-15Rα levels in schizophrenia patients. Furthermore, such insufficiency or lack of IL-15Rα could induce hyperexcitability in the experimental mouse model and schizophrenia patients.

## Data Availability Statement

The original contributions presented in the study are included in the article/Supplementary Material, further inquiries can be directed to the corresponding authors.

## Ethics Statement

The studies involving human participants were reviewed and approved by the Independent Ethics Committee (IEC) of the Beijing Anding Hospital, Capital Medical University, Beijing, China (2015127FS-2). The patients/participants provided their written informed consent to participate in this study. The animal study was reviewed and approved by the Animal Use and Care Committee at Capital Medical University.

## Author Contributions

YH, ZS, and CW were responsible for study design. QB, ZM, and ML were responsible for recruiting the patients, performing the clinical rating, and collecting the samples. YH and ZS were responsible for the assay of IL-15Rα concentration in participants. YH and HW were responsible for the animal experiments. ZS, YH, JY, and CW were involved in the manuscript preparation and providing the funding for the study. AK and WP were involved in the manuscript revision. All authors have contributed to and have approved the final manuscript.

## Conflict of Interest

WP was employed by the company BioPotentials Consult. The remaining authors declare that the research was conducted in the absence of any commercial or financial relationships that could be construed as a potential conflict of interest.

## Publisher’s Note

All claims expressed in this article are solely those of the authors and do not necessarily represent those of their affiliated organizations, or those of the publisher, the editors and the reviewers. Any product that may be evaluated in this article, or claim that may be made by its manufacturer, is not guaranteed or endorsed by the publisher.

## Footnotes

1
http://www.brainspan.org
