# Supplementary material for: Reduced Serum Levels of Soluble Interleukin-15 Receptor α in Schizophrenia and Its Relationship to the Excited Phenotype
Source: Front Psychiatry. 2022 Mar 9;13:842003. doi: 10.3389/fpsyt.2022.842003 (PMC8959406; doi:10.3389/fpsyt.2022.842003)
Supplement: Supplementary file 1 [file Data_Sheet_1.docx]

Supplementary table 1. Comparison of serum IL-15Rα levels in patients with or without antipsychotic treatment at the time of enrollment.

|  | Drug naive (n = 20) | Others (n = 43) | Z | *p* |
| --- | --- | --- | --- | --- |
| IL-15Rα Concentration | 142.78±67.40 | 153.53±112.00 | -0.258 | 0.796 |

Values represent means ± S.D

Supplementary table 2. Associations of the serum IL-15Rα levels and MCCB subscores in schizophrenia patients.

|  | B | Standard coefficient | *t* | *p* |
| --- | --- | --- | --- | --- |
| Speed of processing | 2.188 | 0.176 | 0.657 | 0.516 |
| Attention/vigilance | -0.716 | -0.064 | -0.307 | 0.761 |
| Working memory | 0.810 | 0.077 | 0.334 | 0.741 |
| Verbal learning and memory | -1.351 | -0.107 | -0.497 | 0.623 |
| Visual learning and memory | -0.179 | -0.026 | -0.106 | 0.917 |
| Reasoning and problem-solving skills | -0.428 | -0.045 | -0.194 | 0.848 |
| Social cognition | -0.140 | -0.016 | -0.082 | 0.935 |
